# Prediction of lncRNA functions using deep neural networks based on multiple networks

**DOI:** 10.1186/s12864-023-09578-w

**Published:** 2023-11-09

**Authors:** Lei Deng, Shengli Ren, Jingpu Zhang

**Affiliations:** 1 School of Computer Science and Engineering, Central South University, 410075 Changsha, China; 2 School of Computer and Data Science, Henan University of Urban Construction, 467000 Pingdingshan, China

**Keywords:** Gene ontology, lncRNA functions, PPMI, SDAE, Network representation

## Abstract

**Background:**

More and more studies show that lncRNA is widely involved in various physiological processes of the organism. However, the functions of the vast majority of them continue to be unknown. In addition, data related to lncRNAs in biological databases are constantly increasing. Therefore, it is quite urgent to develop a computing method to make the utmost of these data.

**Results:**

In this paper, we propose a new computational method based on global heterogeneous networks to predict the functions of lncRNAs, called DNGRGO. DNGRGO first calculates the similarities among proteins, miRNAs, and lncRNAs, and annotates the functions of lncRNAs according to its similar protein-coding genes, which have been labeled with gene ontology (GO). To evaluate the performance of DNGRGO, we manually annotated GO terms to lncRNAs and implemented our method on these data. Compared with the existing methods, the results of DNGRGO show superior predictive performance of maximum F-measure and coverage.

**Conclusions:**

DNGRGO is able to annotate lncRNAs through capturing the low-dimensional features of the heterogeneous network. Moreover, the experimental results show that integrating miRNA data can help to improve the predictive performance of DNGRGO.

**Supplementary Information:**

The online version contains supplementary material available at 10.1186/s12864-023-09578-w.

## Background

LncRNA is an RNA molecule that is defined as endogenous molecules with a length of more than 200 nucleotides. More and more biologically-functioning lncRNAs are continually being found in various organisms. LncRNAs are widely involved in animal neurodevelopment, cell cycle regulation, cell regulation, tumorigenesis, and metastasis [1, 2]. Moreover, it is reported that human diseases and cancers are associated with mutations and dysregulations of lncRNAs [3–6]. Thus, identifying functions of lncRNAs has become increasingly important. During those years, a few functions of long non-coding RNAs (lncRNAs) have been annotated by the development of high-throughput next-generation sequencing techniques and lncRNA chip technology [7–9]. There are still a large number of lncRNAs need to be annotated.

Based on biological experiments, biologists can identify functions of lncRNAs through a variety of mechanisms, such as pIgR, CLIP, RAP, etc. However, the experimental characterization of lncRNA functions often costs too much money while there also will be a slow process [10]. Besides some biological methods, recently, several approaches and tools have been designed to identify functions of lncRNAs. Genes with similar expression patterns across multiple conditions usually have close functional relationship or are associated with related biological pathways. Therefore, some researchers determined the lncRNA functions according to the co-expression patterns of genes. Guttman et al. used mouse microarray data and lncRNA-mRNA co-expression data to construct a network to predict the functions of lncRNAs [11]. Liao et al. also used those microarray expression profiles, as well as local information, to annotated functions of 340 lncRNAs, which were concluded by constructing the coexpression of encoding-non-coding [12]. In addition to these local methods, a bichromatic biological network was established to predict the functions of lncRNAs based on coexpression data and protein interaction data by Guo et al. [13]. Recently, Jiang et al. have further proposed a method called LncRNA2Function, which was developed to identify the functions of 9625 lncRNAs by hypergeometric tests [14]. More recently, Zhang et al. have annotated lncRNAs with gene-ontology terminology based on KATZ measures [15]. Functions of lncRNA could be investigated based on integrative features including sequence-derived features such as ORF, nucleotide composition, conservation score, experimental features, etc. The COME method integrated sequence-derived and experimental features to infer the coding potential of lncRNAs [16]. Combining chromatin state data and gene expression patterns, LncRNA Ontology employed the nearest shrunken centroid algorithm to predict the function of lncRNAs [17].

Network learning is a set of techniques that aims to map data structures into latent spaces efficiently. Either for dimension reduction or for exploring semantic content, this type of feature embedding has proved to be robust for node classification. In this study, based on network representations, we developed a novel predictor named DNGRGO, in which we used GO terms as functional annotations for lncRNAs. In this method, we built a global heterogeneous network at first, which contained six networks, namely, lncRNA similarity network, lncRNA-protein association network, protein-protein interaction network, miRNA-lncRNA association network, miRNA-protein network, and miRNA-miRNA co-expression network. Then, we used random walk with restart(RWR) and stacked denoising autoencoder to calculate the low-dimensional features of each node in the network. Finally, we annotated lncRNAs by training an SVM classifier for each GO term based on these compact features and annotations of the protein. To evaluate DNGRGO, we run it on the manually organized independent test set, namely lncRNA2GO-68. Moreover, to illustrate the performance of our method, we compared our experimental results with the three latest methods, KATZGO [15], PLNRGO [18], and BIRWLGO [19]. The experimental results indicate that our method is better than others in terms of F-measure on the independent test set.

## Results

### Benchmark

We evaluated DNGRGO and compared it with other methods through independent validation. However, there was no functional annotation dataset for lncRNAs. Hence, we manually annotated each gene in lncRNA2GO-68 through the sequence, structural information, genomic background, expression, and other information about lncRNAs that had been experimentally verified in the literature (the Additional file 1).

### Evaluation measures

We used the trained SVM model to make predictions for each lncRNA in the independent dataset. Each lncRNA is corresponding to several possible GO terms, and the score of each GO term is between 0 and 1. The higher the score, the more confident the prediction is. Therefore, we need to set a threshold of *t* to determine the final predicted term *p*(*t*). We considered all GO items in each lncRNA which were greater than or equal to *t* as the prediction set *p*(*t*), and each lncRNA manually annotated GO items as the experimental verification set *T*. To measure the performace of predictive methods, we adopted three commonly used measurements, namely precision, recall, and F-measure. For a rank threshold, precision and recall are defined as followings:

1 $$\begin{aligned} Pr_i(t)=\frac{\sum _{f\in O} I(f\in P_i(t)\wedge f\in T_i)}{\sum _{f\in O} I(f\in P_i(t)} \end{aligned}$$

and

2 $$\begin{aligned} Rc_i(t)=\frac{\sum _{f\in O} I(f\in P_i(t)\wedge f\in T_i)}{\sum _{f\in O} I(f\in T_i)} \end{aligned}$$

Where, *O* denotes the data set of the entire gene ontology, and *f* represents a specific GO item in the entire ontology. *I*(*x*) is the indicator function, which is described as:

3 $$\begin{aligned} I(x)=\left\{ \begin{array}{rcl} 1 &{} &{} x=true \\ 0 &{} &{} x=false \end{array} \right. \end{aligned}$$

After prediction, we expected to draw the PR curve by calculating the average precision and recall under different thresholds. A predicted lncRNA corresponds to several possible GO terms, and each GO term corresponds to a probability score. If at least one probability score is greater than or equal to the threshold, we put this lncRNA into the m(t)($\leqq$ N ) dataset. Based on these m(t) lncRNAs, we can calculate the average precision corresponding to each threshold *t*. Then we define the average precision as:

4 $$\begin{aligned} Pr(t)=\frac{1}{m(t)}*\sum \limits _{i=1}^{m(t)}Pr_i(t) \end{aligned}$$

Similarly, we can use the same way to calculate the average recall in the independent test dataset containing *N* lncRNAs. Then the average recall can be defined as:

5 $$\begin{aligned} Rc(t)=\frac{1}{N}*\sum \limits _{i=1}^{N}Rc_i(t) \end{aligned}$$

Different thresholds will lead to different precision and recall. In large-scale data sets, these two indicators are often mutually restrictive. When the threshold is larger, fewer GO terms are predicted of each lncRNA, which can get higher precision, but this will also lead to a lower recall. When the threshold is lower, more GO terms are predicted of each lncRNA, which can get a higher recall rate but also lead to lower precision. To solve this problem, we need to weigh these two indicators (precision and recall) comprehensively, which is to calculate the maximum F-measure for all thresholds. It can be calculated as the following:

6 $$\begin{aligned} F_{max}=\max _{t}(\frac{2*Pr(t)*Rc(t)}{Pr(t)+Rc(t)}) \end{aligned}$$

### Parameter tuning

In our method, we used RWR to extract the structural information of the global network. The RWR algorithm contains a parameter $\alpha$, which denotes the restart probability. The setting $\alpha$ takes the value from 0 to 1. Assuming starting from a certain node, the larger the value of $\alpha$, the greater the probability of returning to the starting node. To validate the influence of its different values, we increased $\alpha$ from 0.1 to 0.9 with step size 0.1. The demonstration shows that the performance is relatively stable when $\alpha$ is set to different values. In the experiment, we chose the restart probability $\alpha$ to be 0.5. After obtaining the topological features of the global network, SDAE was employed to reduce the dimension of features. In the SDAE network, there are many hyperparameters to be tuned. We set the same values as Cao et al.’s research [20]. The number of layers of the entire network was set to 5, and the number of nodes in each layer denoted as *M* equals [36863-10000-3000-1000-512].

**Fig. 1:**
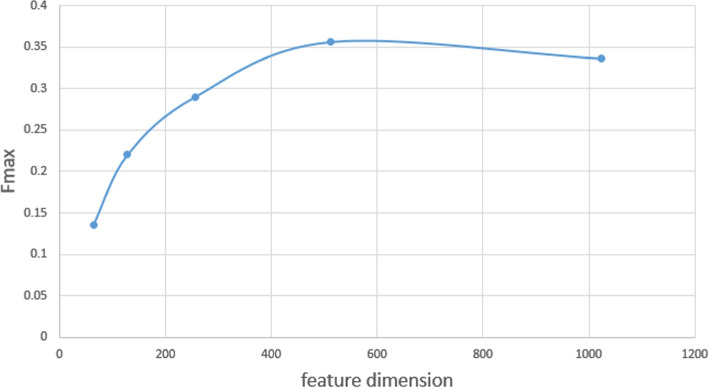
Influence of different feature dimension for function prediction

We extracted features of different dimensions and calculated $F_{max}$ on the lncRNA2GO-68 dataset for evaluation, because features of different dimensions may affect the prediction performance of DNGRGO. As shown in Fig. 1, $F_{max}$ increases first and then decreases gradually when the dimension increases. It comes to the max value when the dimension equals 512. Hence, we finally reduced the high-dimensional features to 512 dimensions and entered them as input to the classifier.

### Effect of integrating miRNA data

There have been several methods for investigating functions of lncRNAs through integrating multiple data sources, such as KATZLGO, BiRWLGO, PLNRGO. Compared with these methods, DNGRGO has newly added miRNA data. To validate the effectiveness of miRNA data, we evaluated DNGRGO on two different network configurations including: the network without miRNAs (miRNA-miRNA similarities, miRNA-protein interactions, and miRNA-lncRNA associations removed) and the entire network. These two configurations of DNGRGO were tested on the lncRNA2GO-68 dataset for precision, recall, and $F_{max}$. As shown in Table 1, the $F_{max}$ score is 0.356 for the entire network, and 0.306 for the network without miRNAs. The results show that the entire network with integrated miRNA data can significantly better predict functions of lncRNAs than the network without integrated miRNAs .

**Table 1:** Performance comparision on two different network configurations: the network without miRNAs and the entire network

Method	Recall	Precision	Fmax
the entire network	0.395	0.324	0.356
the network without miRNAs	0.515	0.218	0.306

### Performance compared with other methods

At present, the most commonly used method for predicting functions of lncRNAs based on co-expression is “guilt-by-association”. The conclusion is that if lncRNAs and the coding genes have similar expression patterns, they have similar functions [21]. The KATZ measure assigns different weights to neighboring nodes, giving larger weights to short paths and smaller weights to long paths. KATZLGO builds a global network of lncRNA and protein, then uses the KATZ measure to calculate the correlation scores between each pair of genes, and finally selects the GO term corresponding to the protein with the high correlation score as the functional annotation of lncRNAs [15]. In the BiRWLGO method, a global heterogeneous network of lncRNA and protein is constructed, and a double random walk is performed to calculate the probability scores between all lncRNA-protein pairs. A higher probability indicates a higher degree of association between the pair of genes. Then, the prediction of lncRNA functions can be achieved through the adjacent protein annotated with the GO terms [19]. Same as the first two methods, PLNRGO first constructs a heterogeneous network of lncRNA-proteins, then uses random walks to extract network features, and uses SVM to predict the functions of lncRNAs [18].

**Fig. 2:**
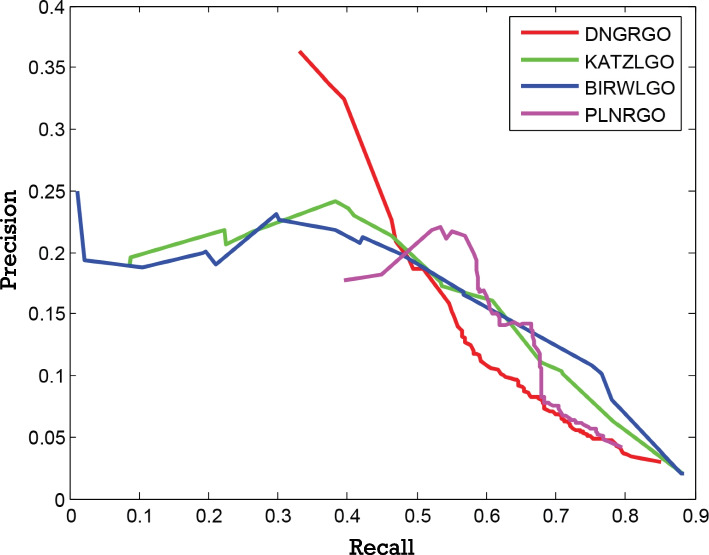
The precision-recall curve is used to estimate the overall performance

**Fig. 3:**
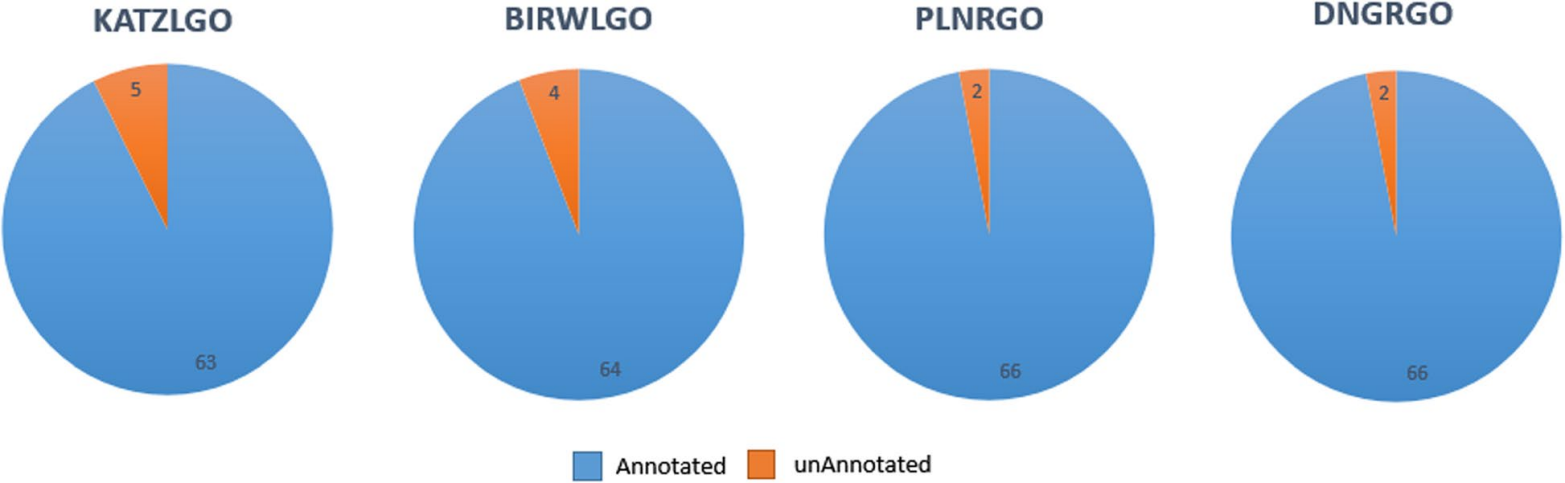
The numbers of lncRNAs that are annotated correctly by different methods, respectively

In this paper, our method DNGRGO is compared with the three methods in precision, recall, and $F_{max}$. The detailed comparison results are shown in Table 2. Moreover, the precision-recall curves of different methods are plotted in Fig. 2. As shown, DNGRGO achieves the highest $F_{max}$ score of 0.356, which performs better than the other three methods. Besides $F_{max}$, our method also gains the highest score of precision. Besides, the number of lncRNAs correctly annotated is shown in Fig. 3. 66 lncRNAs of the manually organized 68 lncRNAs are correctly annotated by our method and PLNRGO, KATZLGO and BiRWGO follow with the numbers of 63 and 64.

**Table 2:** Performance comparison with other methods on the lncRNA2GO-68 dataset

Method	Recall	Precision	Fmax
DNGRGO	0.395	0.324	0.356
KATZLGO	0.382	0.241	0.297
BiRWLGO	0.422	0.212	0.282
PLNRGO	0.535	0.220	0.312

### Case study

To further illustrate the performance of our prediction method, we used the prediction results of NEAT1 as a case. NEAT1 is a long non-coding RNA that is critical to speckle integrity. Studies of gain-of-function or loss-of-function in C2C12 cells have shown that NEAT1 promotes myoblast proliferation but inhibits myoblast differentiation and fusion [22]. NEAT1 is downregulated in acute promyelocytic leukemia, where it promotes leucocyte differentiation [23, 24]. The results show that the Wnt signaling pathway is activated by knockdown inactivation of NEAT1. And the Wnt signaling pathway is related to many important cell functions, such as cancer stem cells [25]. NEAT1 knockdown cells produced smaller tumors, demonstrating that NEAT1 promotes tumor growth in vivo [26]. We used the DNGRGO method to predict 158 GO annotations for NEAT1, and then we ranked the GO terms in descending order of predicted scores, of which the first 30 GO terms are listed in Table 3. As predicted, many of them are related to metabolism, which are closely related to the development of cancer, such as GO: 0019222 (regulation of metabolic process), GO: 0044237 (cellular metabolic process), GO: 0032946 (positive regulation of Mononuclear cell proliferation), GO: 0051493 (regulation of lipid metabolic process), GO: 0050794 (regulation of cellular process), GO: 0046434 (organophosphate catabolic process). There are also a large number of GO terms related to signal channels, such as GO: 0007165 (signal transduction), GO: 0005102 (signaling receptor binding), GO: 0007167 (enzyme-linked receptor protein signaling pathway), GO: 0009755 (hormone-mediated signaling pathway), GO: 0016055 (Wnt signaling pathway).

**Table 3:** The top 30 predicted BP terms for lncRNA NEAT1 by DNGRGO

Rank	GO term	GO name
1	GO:0007166	cell surface receptor signaling pathway
2	GO:0017076	purine nucleotide binding
3	GO:0016192	vesicle-mediated transport
4	GO:0019222	regulation of metabolic process
5	GO:0044237	cellular metabolic process
6	GO:0008270	zinc ion binding
7	GO:0007165	signal transduction
8	GO:0003676	nucleic acid binding
9	GO:0046907	intracellular transport
10	GO:0016197	endosomal transport
11	GO:0009987	cellular process
12	GO:0000166	nucleotide binding
13	GO:0007159	leukocyte cell-cell adhesion
14	GO:0015711	organic anion transport
15	GO:0005102	signaling receptor binding
16	GO:0006936	muscle contraction
17	GO:0009991	response to extracellular stimulus
18	GO:0007167	enzyme linked receptor protein signaling pathway
19	GO:0046434	organophosphate catabolic process
20	GO:0009755	hormone-mediated signaling pathway
21	GO:0050794	regulation of cellular process
22	GO:0030522	intracellular receptor signaling pathway
23	GO:0030518	intracellular steroid hormone receptor signaling pathway
24	GO:0051493	regulation of cytoskeleton organization
25	GO:0051716	cellular response to stimulus
26	GO:0019216	regulation of lipid metabolic process
27	GO:0032946	positive regulation of mononuclear cell proliferation
28	GO:0016055	Wnt signaling pathway
29	GO:0007154	cell communication
30	GO:0046942	carboxylic acid transport

## Discussion and conclusion

Many studies have shown that lncRNA plays an important role in cell function. However, the functional annotation and prediction of lncRNAs have become a considerable challenge due to the non-conservative primary sequence and unstable secondary structure of lncRNAs. In our study, we proposed a deep neural network-based method, DNGRGO, which predicts the GO annotation of lncRNAs by extracting low-dimensional feature vectors from the global network and training a SVM classifier. Based on the manually annotated lncRNA2GO-68 dataset, we assessed the performance of DNGRGO independently. Experimental results show that DNGRGO scores 0.356 and 0.324 on $F_{max}$ and precision, respectively, far higher than the other three methods. In addition, our experiments show that integrating miRNA data into the network can effectively improve the performance of lncRNA’s functional prediction. In the end, we believe that DNGRGO, as a supplement to biological protocols, will further enrich the study of lncRNA functions.

## Methods

**Fig. 4:**
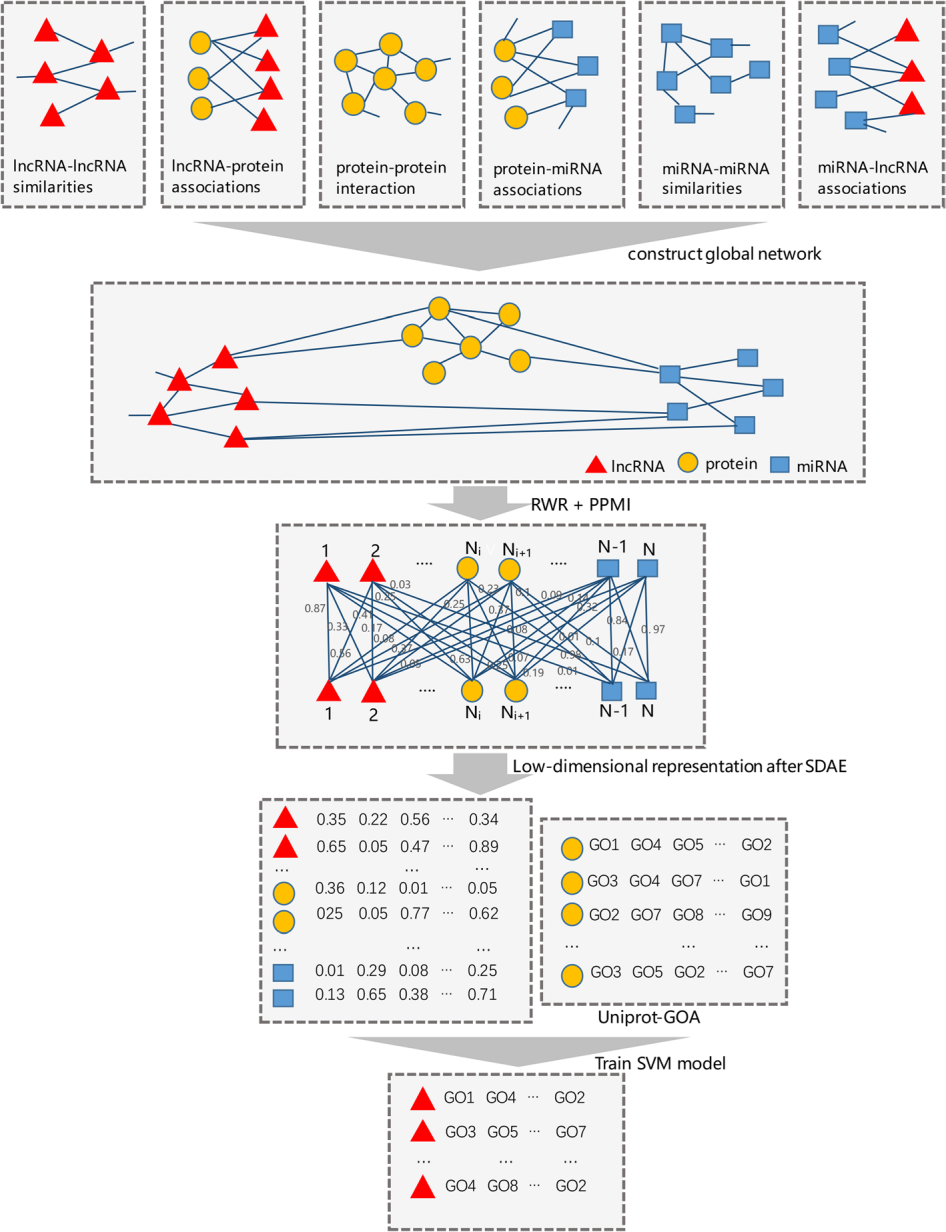
Flowchart of DNGRGO. It consists of four steps: (A) Build the global heterogeneous network composed of six component networks. (B) capture the topological feature of each node through running RWR algorithm on the global network, and calculate the PPMI according to these features. (C) Obtain the low-dimensional feature vectors through SDAE. (D) SVM models are built for different gene ontology terms

To predict the potential functions of lncRNAs, we proposed a new model named DNGRGO, which consisted of four steps, as shown in Fig. 4. First, we integrated the six networks into a large global network, which contained the protein-protein interaction network, protein-lncRNA association network, lncRNA similarity network, miRNA-miRNA co-expression network, miRNA-protein association network, and miRNA-lncRNA association network. Then we used the random walk with restart (RWR) to extract graph structure information and calculated the positive point of mutual information (PPMI) matrix. To extract low-dimensional features from the PPMI matrix, we used a stack denoising autoencoder to reduce the dimensions. Finally, we trained the SVM model based on topological features and annotation of protein-coding genes, and applied them to annotate the potential functions of lncRNAs.

### Materials

#### lncRNA co-expression similarities

All human lncRNA co-expression data is obtained from NONCODE2016 database [27]. It contains the expression profiles of 90062 human lncRNAs. We calculated Pearson’s correlation coefficient (PCC) between each pair of lncRNAs to represent the co-expression similarity of lncRNAs. The Ensemble ID list of lncRNA genes and the co-expression similarities are provided in the Additional file 2 and 3, respectively.

#### protein-protein interactions

We downloaded protein-protein interaction data from the STRING database V10.0 [28]. The STRING database is a tool for searching for the relationship between genes and proteins. It contains 2031 species, 9,637,763 proteins, and 1,380,838,440 interactions. In the end, we obtained 17867232 PPI relationships from the database. The Ensemble ID list of coding genes and the PPIs are provided in the Additional files 4 and 5, respectively.

#### lncRNA-protein associations

To obtain lncRNA protein-data for building a global network, we first downloaded all human lncRNA genes and protein-encoding genes from the GENCODE database of release 24 [29]. After screening, a total of 15941 lncRNAs and 20284 proteins were extracted. To build the lncRNA-protein associations, we combined three data sources, which are as follows:

I. Co-expression data from COXPRESdb [30]. COXPRESdb is a database that provides co-expression information of 11 animal species. In COXPRESdb, we got a pre-processed lncRNA-protein co-expression dataset, which mainly refers to the Pearson correlation coefficient between human gene pairs. The specific calculation is as follows:

7 $$\begin{aligned} S(l,p)=1-\prod _{n=1}^N(1-S_n(l,p)) \ \ if\ S_n(l,p)>0 \end{aligned}$$

where *S*(*l*, *p*) represents the overall correlation between lncRNA *l* and the protein-coding gene *p*, $S_n(l,p)$ is the correlation score between *l* and *p* in the local data set *n*, and *N* is the number of *l*-*p* gene pairs with positive correlation scores. We only considered positive correlation scores and removed negative correlation scores of gene pairs.

II.Co-expression data from ArrayExpress [31] and GEO [32]. Jiang et al. [14]processed the co-expression data in these two databases and built a web server for us to download. We used the Pearson correlation coefficient to indicate the degree of association between lncRNA and protein.

III. LncRNA-protein interactions from NPinter 3.0 [33]. The lncRNA-protein interactions of ’Homo sapiens’ were downloaded from the NPinter database, which contains 491416 ncRNA interaction data with other biomolecules, and these data have been experimentally verified. If there are lncRNA-protein pairs in the interaction data set, we can set their interaction scores to 1, otherwise set to 0.

The integrated lncRNA-protein associations are provided in the Additional file 6.

#### miRNA-miRNA co-expression similarities

The miRNA expressions involving 638 miRNAs (the Additional file 7) are curated from the mimiRNA [34] database. We calculated the PCC score of each pair of miRNAs as the co-expression similarity of miRNAs (the Additional file 8).

#### miRNA-protein interactions

We downloaded the known miRNA-protein associations from RAID V2.0 [35], which covered more than 60 species and had more than 5.27 million RNA-related interactions, including more than 1.2 million RNA-protein interaction data. Then, we evaluate the reliability of each RNA interaction based on the comprehensive confidence score. After preprocessing, we finally obtained 2133 miRNA-protein associations (the Additional file 9).

#### miRNA-lncRNA associations

We downloaded miRNA-lncRNA associations from the starBase database [36], which provided the experimentally confirmed miRNA-lncRNA interactions. After removing the redundant items, we collected 4983 miRNA-lncRNA associations (the Additional file 10).

### Construct the global network

Different types of biological data can be integrated to construct networks of biological interactions, thereby correlating potential function. Usually, combining more interactions can be effective for the lncRNA annotations. The theoretical basis for this conclusion is that interacting protein, and lncRNAs tend to have the same or similar functions [37]. In addition, if genes have transcripts with similar expression patterns, they may share related biological pathways or have similar functions. Therefore, integrating multiple biological datasets can help annotate the functions of lncRNAs. In our work, we annotate the functions of lncRNAs by integrating six-component networks. Let *L*, *P*, *M*, *LP*, *LM*, *PM* represent the adjacency matrices for lncRNA similarity network, protein-protein interaction network, miRNA-miRNA co-expression network, lncRNA-protein association network, lncRNA-miRNA association network, protein-miRNA association network, respectively. In addition, we represent the global network as the following:

8 $$\begin{aligned} G= \left[ \begin{array}{ccc} L &{} LP &{} LM \\ LP^{\textrm{T}} &{} P &{} PM \\ LM^{\textrm{T}} &{} PM^{\textrm{T}} &{} M \end{array}\right] \end{aligned}$$

Where, T in $LP^{\textrm{T}}$, $LM^{\textrm{T}}$, $PM^{\textrm{T}}$ denotes the transpose.

### Obtain vector representations of nodes

To capture the topological information of the nodes in the global heterogeneous network, we adopted the DNGR model to obtain the vector representations of nodes [20]. In DNGR, the random walk with restart (RWR) algorithm was employed to extract the contextual information for the nodes. RWR considers not only the local but also the global structural information of the network. It measures the transition probability of the nodes on the graph, and the final distribution can be used to find out the correlations among the nodes. In the formula, *G* represents a weighted adjacency matrix, which is the global heterogeneous network we build. And *A* represents the transition matrix, and the sum of each column in the transition matrix is 1. Matrix A can be obtained by applying the column normalization of *G*. And, each entry $A_{i,j}$ in *A* represents the probability of walking from node *i* to node *j*, which is given by:

$$\begin{aligned} {A_{{i,j}}=\frac{{G_{{i,j}}}}{{{\mathop { \sum }\nolimits _{{k}}{G_{{k,i}}}}}}} \end{aligned}$$

RWR can be formulated as following recurrence relation:

9 $$\begin{aligned} P_k=\alpha *P_{k-1}*A+(1-\alpha )*P_0 \end{aligned}$$

Where, $P_0$ represents the identity matrix, each column in the matrix is a 1-hot code, that is, the *j*-th item is 1, and the other items are 0. $P_k$ represents the matrix obtained after *k* steps, and each row of the matrix represents the association between the current node and other nodes in the graph. Starting from a certain node in the graph, each step faces two choices, randomly selects neighboring nodes or returns to the starting node. And $\alpha$is the probability of restart, means the probability of returning to the original node and restarting the random surfing procedure. 1-$\alpha$ represents the probability of moving from the current node to a neighbor node. After multiple iterations, the probability distribution reaches a plateau, which is called the ’diffusion state’. Intuitively, the closer two nodes are, the more intimate the relationship they should have. This means they may have similar functions. Based on the matrix of the diffusion state, we refactor a vector representation of all the nodes in the global network by computing the PPMI matrix.

The PPMI matrix obtained from above approaches is highly dimensional when the network is large. As such, these features cannot be readily used for prediction. To extract the high-quality low-dimensional vector representation for nodes from the PPMI matrix, we employed stacked denosing autoencoder (SDAE) to generate compress low-dimensional vectors.

The stacked denosing autoencoder is based on the automatic encoder. We used the backpropagation algorithm to make the target value equal to the input value. An autoencoder can be divided into two parts: the encoder and the decoder. The autoencoder first receives the input vector, maps it to a low-dimensional latent representation space through a mapping function $f_{\theta _1}(.)$, and then reconstructs the latent representation space into the original input vector by a reconstruction function $g_{\theta _2}(.)$. It is assumed that $f_{\theta _1}\left( {x} \right) = \sigma \left( W_{1}x+b_{1} \right)$ and $g_{\theta _{_{2}}}\left( x \right) = \sigma \left( W_{2}y+b_{2} \right)$, where $\sigma \left( . \right)$ denotes the activation function, $\theta _{1}=\left\{ W_{1} ,b_{1}\right\}$ and $\theta _{2}=\left\{ W_{2} ,b_{2}\right\}$ are the weights in the encoder and the decoder, respectively. The aim is to find the optimal $\theta _1$ and $\theta _2$ by minimizing the loss function:

10 $$\begin{aligned} \underset{\theta _1,\theta _2}{\textrm{min}}\sum \limits _{i=1}^nL(x^{(i)},g_{\theta _2}(f_{\theta _1}(\tilde{x}^{(i)}))) \end{aligned}$$

**Fig. 5:**
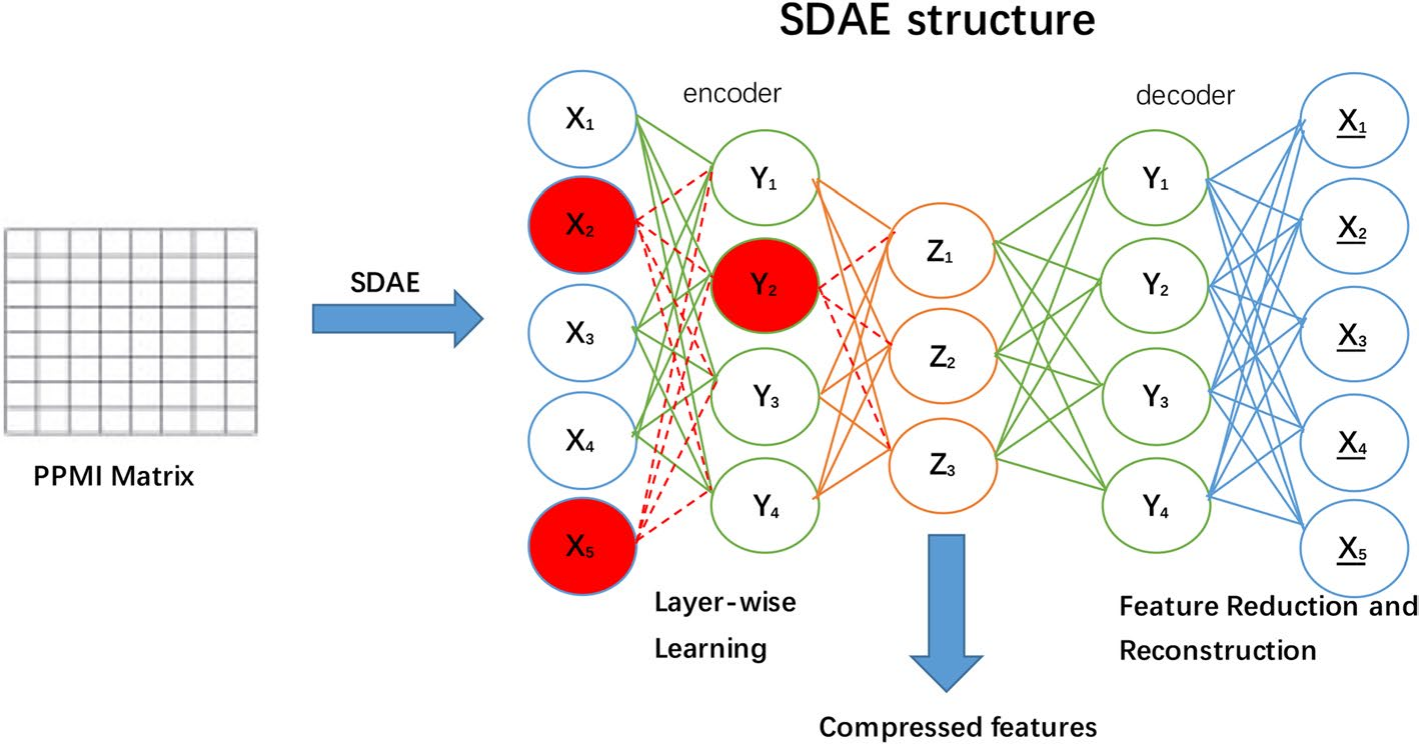
Low-dimensional features are extracted from the middle layer of SDAE. It performs two actions: an encoding step, followed by a decoding step

Where, *L* is the standard squared loss. As shown in Fig. 5, the PPMI matrix, which is denoted as $x_{i}$, is taken as the input into the SDAE model. $y_{i}$ denotes the learned representations in the first layer, and $z_{i}$ represents the learned representations in the second layer. We train the model by minimizing the loss function, which can be optimized by the standard back-propagation algorithm. When the loss function comes to the minimum, we can extract the low-dimensional features from its bottleneck layer.

### Train the SVM models

In this paper, we build a support vector machine (SVM) classifier for each GO term. And the compressed low-dimensional representations calculated in the previous step are taken as the input features. We download the annotations of proteins from GOA-PDB [38]. The proteins with length between 50 and 100 amino acids are clustered with sequence similarity greater than 90%. For each cluster, only one protein is selected as a representation. In these representations, we deleted the proteins without at least a manually assigned (non-IEA) GO terms. For each GO annotation, the protein-GO pairs with the protein having the GO annotation are considered positive samples, and the protein-GO pairs with the protein not having the GO annotation are considered negative samples. Generally, the protein-Go pairs in the positive set are more than those in the negative set. To generate a balanced training data set, We randomly select the negative samples as many as positive samples. Based on the training set consisting of the positive and negative samples, a SVM classifier is built for a specific GO term.

### Supplementary information

**Additional file 1.** The functional annotation dataset.

**Additional file 2.** The Ensemble ID list of lncRNA genes.

**Additional file 3.** The co-expression similarities of lncRNAs.

**Additional file 4.** The Ensemble ID list of coding genes.

**Additional file 5.** The protein-protein interactions.

**Additional file 6.** The lncRNA-protein associations.

**Additional file 7.** The miRNA ID list.

**Additional file 8.** The co-expression similarities of miRNAs.

**Additional file 9.** The miRNA-protein interactions.

**Additional file 10.** The miRNA-lncRNA associations.

## Data Availability

The data sets of DNGRGO are freely available in Additional information in the article.

## Abbreviations

GO: Gene Ontology
PPI: Protein-Protein interaction
SDAE: Stacked Denoise Autoencoder
PPMI: Positive point of mutual information
PCC: Pearson’s correlation coefficient
PR: Precision-Recall
